# Lithium-ion electrolytic substrates for sub-1V high-performance transition metal dichalcogenide transistors and amplifiers

**DOI:** 10.1038/s41467-020-17006-w

**Published:** 2020-06-24

**Authors:** Md Hasibul Alam, Zifan Xu, Sayema Chowdhury, Zhanzhi Jiang, Deepyanti Taneja, Sanjay K. Banerjee, Keji Lai, Maria Helena Braga, Deji Akinwande

**Affiliations:** 1 Microelectronics Research Center, Department of Electrical and Computer Engineering, The University of Texas, Austin, TX 78758 USA; 2 Department of Physics, The University of Texas, Austin, TX 78712 USA; 3 LAETA, Engineering Physics Department, Engineering Faculty, University of Porto, R. Dr. Roberto Frias s/n, 4200-465 Porto, Portugal

**Keywords:** Engineering, Materials science, Nanoscience and technology, Physics

## Abstract

Electrostatic gating of two-dimensional (2D) materials with ionic liquids (ILs), leading to the accumulation of high surface charge carrier densities, has been often exploited in 2D devices. However, the intrinsic liquid nature of ILs, their sensitivity to humidity, and the stress induced in frozen liquids inhibit ILs from constituting an ideal platform for electrostatic gating. Here we report a lithium-ion solid electrolyte substrate, demonstrating its application in high-performance back-gated n-type MoS_2_ and p-type WSe_2_ transistors with sub-threshold values approaching the ideal limit of 60 mV/dec and complementary inverter amplifier gain of 34, the highest among comparable amplifiers. Remarkably, these outstanding values were obtained under 1 V power supply. Microscopic studies of the transistor channel using microwave impedance microscopy reveal a homogeneous channel formation, indicative of a smooth interface between the TMD and underlying electrolytic substrate. These results establish lithium-ion substrates as a promising alternative to ILs for advanced thin-film devices.

## Abstract (web summary)

Electrostatic gating of 2D transistors with ionic liquids presents intrinsic limitations. Here, the authors demonstrate n-type MoS_2_ and p-type WSe_2_ transistors on a lithium-ion solid electrolyte substrate, displaying sub-threshold values approaching the ideal limit of 60 mV/dec and complementary amplifier gain of 34 with 1 V supply.

## Introduction

**Fig. 1:**
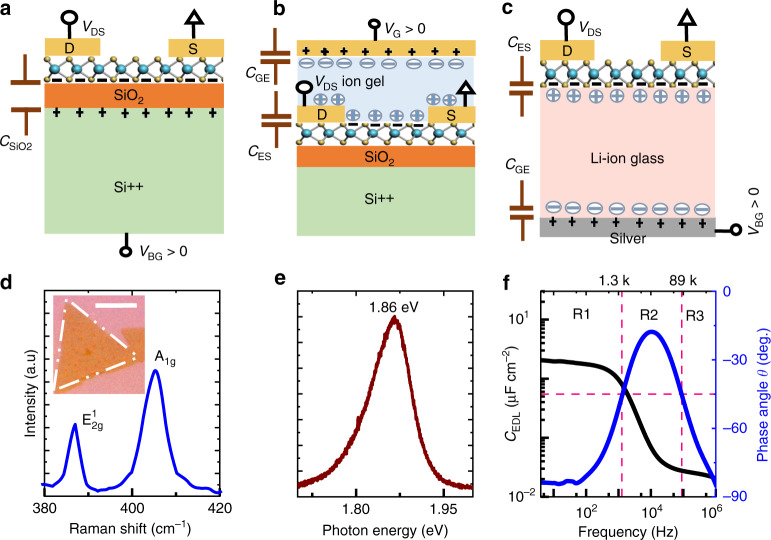
Device operation, material and substrate characterization of monolayer MoS_2_ on Li-ion glass. Schematic illustration of the working principle of **a** solid dielectric-gated FET, **b** ion gel-gated FET and, **c** Li-ion glass-gated FET. The sketches are shown for positive gate bias. The circles and the arrows in the connection schemes represent the bias and ground, respectively. **d** Raman spectrum of the monolayer CVD MoS_2_ transferred onto glass substrate. Inset shows dark-field optical micrograph of the transferred CVD MoS_2_. The boundary of the flake is marked with white dash-dotted lines to aid visualization. Scale bar is 10 µm. **e** Photoluminescence spectrum of the same flake. **f** Capacitance/Phase angle vs. frequency of Li-ion glass substrate with Ni (20 nm) as both top and bottom electrode. The frequency spectrum can be divided into three distinct regions: (i) R1 where the EDL is formed, (ii) R2 where ion migration dominates, and (iii) R3 where the bulk Li-ion glass works as a dielectric.

Field-effect transistors (FETs), wherein charge carrier modulation occurs via gating through the formation of electric double layers (EDLs), have been studied for various classes of channel materials and electrolytes over the past few decades^1–6^. In addition to realizing electric double layer transistors (EDLTs)^7–9^, EDL has also shown success in modulating superconductor-insulator transition^10,11^, electrically induced ferromagnetism^12^, thermoelectric properties^13,14^, and mimicking biological synaptic functions^15,16^. An EDLT comprises of two EDLs, forming at the interfaces of the electrolyte (see Fig. 1a–c): the first one at the metal gate and electrolyte interface (GE interface) and the second one at the interface of electrolyte and semiconductor channel (ES interface). When a positive bias is applied on the gate electrode, positive mobile ions (inorganic solid electrolytes are often cationic conductors) in the electrolyte get repelled from the GE interface, leaving a layer of negatively charged counter-ions at the interface, thereby forming the GE EDL to realign the Fermi levels of gate electrode and electrolyte. In case of ES interface, the positive mobile ions driven away from the GE interface accumulate at the ES interface and in turn induce electrons in the semiconductor channel, hence forming the ES EDL. Analogously, the application of a negative bias on the gate electrode leads to the accumulation of holes in the channel. The distance between the participating opposite charges in an EDL is around 3–8 Å (depending on the ionic concentration, temperature, and dielectric constant)^17,18^, and this sub-nanometer parallel-plate capacitor gives rise to extremely high capacitances, which can lead to a large carrier density (>10^14^ cm^−2^) even at very low gate voltages^1^. With conventional dielectrics, accumulation of such high carrier densities would require either extremely high gate voltages or an ultra-thin dielectric. However, the low breakdown field of bulk dielectrics (<10 MV cm^−1^ for SiO_2_) limits the applicability of the former^19^, while the latter makes the transistor highly susceptible to excessive gate leakage currents.

Ionic liquids are the most extensively studied electrolytes for EDLTs for semiconductors till date, where the high mobility and diffusivity of the ions make them suitable for faster operation^1,20^. However, the usage of ionic liquid presents several issues: (i) it is not suitable for surface characterization studies, as it covers the surface of the active region, (ii) it is not viable for precise or practical transistor studies due to its liquid nature, (iii) some ionic liquids are sensitive to humidity, and (iv) channel material can be stressed or damaged once the liquid is frozen at low temperatures. Ion gel, the solid counterpart of ionic liquid, overcomes some of these limitations, but is still susceptible to humidity and lacks a well-defined physical size^9,21^. Recently, various air-stable solid-state electrolytes (SSEs) have been developed, thanks to the extensive progress in battery technology. Li- and Na-ion-based SSEs have already been used to investigate charge carrier tuning in graphene^22,23^. Gate tunable insulator-to-metal transition has also been studied in MoS_2_ with a LaF_3_-based solid electrolyte^24^. Recently, electrostatic gating in WSe_2_ FET with a Li-ion-based solid electrolyte has been reported with limited characterization and analysis, which are essential for understanding the principle of electrolytic gating^25^. As such, a detailed and comprehensive study of transition metal dichalcogenide (TMD) transistors based on solid electrolytes is yet to be conducted.

In this work, we systematically investigate the transport properties of both n-type (MoS_2_) and p-type (WSe_2_) TMD back-gated transistors fabricated on Li-ion glass substrate, which works both as the gate dielectric and the supporting substrate. Unlike liquid electrolyte gating, we obtain only unipolar conduction for the TMDs (n-type for MoS_2_ and p-type for WSe_2_)^1,20^. All the transistors operate under enhancement mode (e-mode) with a small threshold voltage, a desirable feature. For the best devices, a near-ideal sub-threshold swing (SS) of 60 mV/dec (WSe_2_) and 64 mV/dec (MoS_2_) are observed at room temperature with decent ON/OFF ratios above 10^6^. The output characteristics show a relatively high ON current, Ohmic-like contacts and current saturation. Subsequently, a CMOS inverter amplifier is realized by connecting the n-type and p-type FETs, affording a high voltage gain (~34 V/V), which is the highest gain reported so far with a 1 V power supply. Finally, we investigate the microscopic evolution of local conductivity in the MoS_2_ channel by the microwave impedance microscopy (MIM) technique. Within the mesoscopic length scale (~100 nm), the formation of conductive channel is found to be spatially uniform, in contrast to the observation of strong inhomogeneity in MoS_2_ FETs on conventional SiO_2_/Si substrate^26^.

## Results

### Glass substrate and material characterization

To realize EDLT with TMDs, a double-sided polished glass sheet (150 µm thick, from Ohara Corp.), made of a NASICON type crystal structure containing lithium ions (Li ions) as mobile charge carriers, is used as a solid electrolyte substrate. This air-stable, high-temperature compatible and flat (average roughness ~0.93 nm, Supplementary Fig. 1) substrate is referred to as lithium-ion containing glass or simply Li-ion glass throughout the text. A 3D drawing of a prototypical Li-ion glass EDLT is shown in Supplementary Fig. 2, where silver coating is used as the back-gate metal. As described previously, the two EDLs in a Li-ion glass EDLT are illustrated in Fig. 1c. The major physical difference in EDL formation between an ion gel-gated and Li-ion glass-gated transistor is that the ion gel spreads on the top (Fig. 1b), thereby impeding any surface probe experiments in contrast to the glass, which is underneath the channel. Like EDL formation in ion gel and in Li-ion glass, conventional dielectrics work via electrostatic action, seeking dynamic alignment of the Fermi levels of the materials in electrical contact, leading to uniformly distributed charges of opposite polarity on either side of the dielectric as depicted in Fig. 1a. Although, electrostatic field effect is key to the realization of EDLTs, in some cases, especially at high voltage, Li ions may intercalate into the TMD and cause electrochemical doping^27,28^. However, no electrochemical action or Li intercalation is observed within the extended gate voltage range (*V*_BG_ = −3 V to +3 V), confirmed from the absence of redox-reactions (characterized by the presence of *I* vs *V* ‘duck’-shaped curve) for several devices studied in this work.

Initial studies began with single-layer MoS_2_ that is chemical vapor deposition (CVD)-grown on SiO_2_/Si and subsequently transferred onto Li-ion glass substrate using the conventional poly (methyl methacrylate) (PMMA) based wet-transfer method (Supplementary Fig. 3). The quality and integrity of the transferred material was verified with Raman and Photoluminescence (PL) spectroscopy as shown in Fig. 1d, e, respectively. Peak separation between E^1^_2g_ and A_1g_ is ~18.5 cm^−1^, which is typical for a single layer^29^. The full width at half maximum (FWHM) for in-plane (E^1^_2g_) and out of plane (A_1g_) Raman modes are <3 cm^−1^ and <6 cm^−1^, respectively, which signifies good crystalline quality of the transferred material^30^. The PL peak of the same flake is located at 1.86 eV and the FWHM is ~80 meV, similar to the reported values in literature for good-quality materials^31^. As the optical contrast of ultra-thin TMD is not easily distinguishable against the underlying substrate in bright field, we used dark-field optical microscopy to identify the monolayers. The capacitance (*C*_EDL_) of the Li-ion glass is measured with an LCR meter (HIOKI IM 3536) in the frequency range of 4 Hz- 1 MHz with an AC signal of 100 mV. At 4 Hz, the phase angle reaches close to −83° with a capacitance value of 2 µF cm^−2^ (Fig. 1f). By using quasi-static capacitance measurement (Keysight B1500), we obtain capacitance values with an average of ~2.15 µF cm^−2^ (Supplementary Fig. 4). In this work, we use 2.10 µF cm^−2^, the average of the quasi-static and low-frequency capacitances, as the value of *C*_EDL_. As the quantum capacitance of TMDs is an order of magnitude higher ($C_{\mathrm{Q}} = \frac{{q^2m \ast }}{{\pi \hbar ^2}}$, therefore *C*_Q,MoS2_ ~ 38 µF cm^−2^ and *C*_Q,WSe2_ ~ 30 µF cm^−2^)^32–34^ than *C*_EDL_, we can neglect the quantum capacitance. We note that this value of *C*_EDL_ is close to the value obtained for a fluoride-ion solid electrolyte capacitor^24^. The equivalent oxide thickness (EOT) calculated from the effective capacitance value is ~1.64 nm. Similar to ionic liquid/gel^35,36^, the frequency (*f*) spectrum can be divided into three distinct regions: (i) R1 (*f* < 1.3 kHz) where EDL is formed, (ii) R2 (1.3 kHz < *f* < 89 kHz) where ion migration dominates, and (iii) R3 (*f* > 89 kHz) where the bulk Li-ion glass behaves like a conventional dielectric (Supplementary Fig. 5). By utilizing the frequency-dependent impedance data of Fig. 1f, a room temperature Li ion conductivity (factor affecting device speed, see Supplementary Note 1) of ~0.22 mS cm^−1^ (Supplementary Fig. 6), as expected for Li-ion glass^37^, has been obtained; the liquid electrolytes, alternatively, have conductivities of the order of 1 mS cm^−1^^38^.

### Transistor properties of n-type MoS_2_

**Fig. 2:**
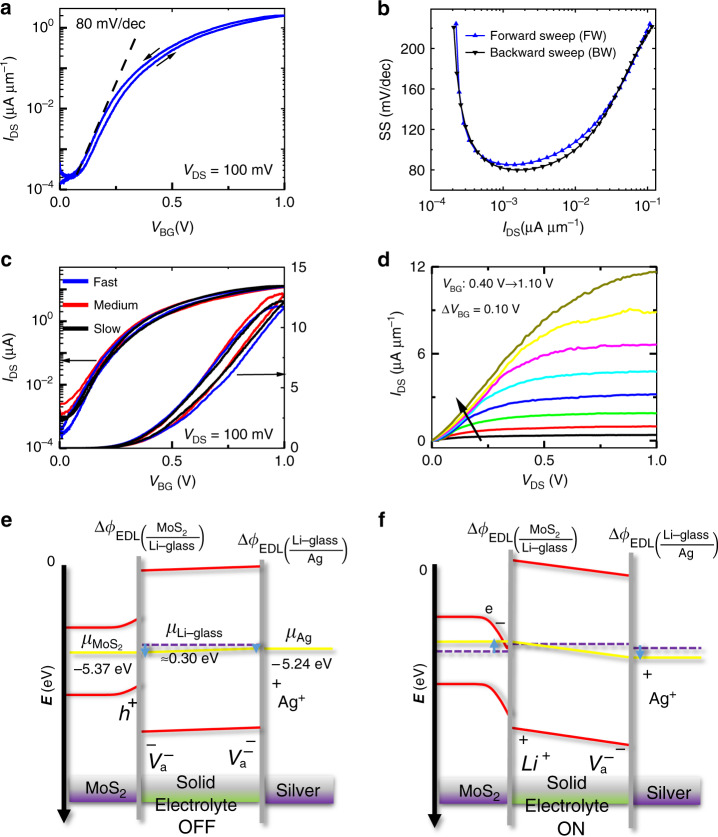
Electrical transport characteristics of monolayer CVD MoS_2_ transferred onto Li-ion glass. **a** Transfer characteristics of a back-gated CVD MoS_2_ FET (*L* = 1 µm, *W* = 5 µm). *V*_TH_ is ~0.4 V. **b** SS vs. *I*_DS_ for the same FET. SS calculated at points in the sub-threshold region of **a**. SS_min_ for forward and backward sweeps are ~85 mV/dec and 80 mV/dec, respectively. **c** Transfer characteristics at different gate sweeping speeds. Sweeping rates for fast, medium, and slow sweeps are 44 mV s^−1^, 9 mV s^−1^, and 1 mV s^−1^, respectively. **d** Output characteristics of the FET at various back-gate voltages. **e**, **f** Schematics illustrating the band diagram for ON/OFF states of MoS_2_ FET. Purple dashed and yellow solid lines represent the initial and final states of the chemical potentials, respectively (see Supplementary Fig. 16 for further details).

An FET device (*L* = 1 µm, *W* = 5 µm) was fabricated on the transferred monolayer CVD MoS_2_ by patterning source-drain electrodes using e-beam lithography (EBL) and contact metal (Ni/Au 20 nm/30 nm) evaporation followed by lift-off. The channel is then defined with another e-beam lithography followed by Ar/Cl_2_ plasma etching. Both the forward and backward sweeps in the transfer characteristics (Fig. 2a) are in very close agreement with each other resulting in a small hysteresis (< 70 mV). The small hysteresis can be attributed to the dielectric nature of the electrolyte, allowing for fast short-range displacement ionic currents with back-gate voltage, even at a sweeping speed of 9 mV s^−1^. We note an anticlockwise hysteresis in the gate transfer characteristics, which might be due to the displacement current caused by mobile ions, whereas the clockwise hysteresis in conventional dielectric-gated FET is usually caused by charged trapping at the channel interface^39^. The effect of the high capacitance of the EDLs formed in the solid electrolyte is reflected in the transfer characteristics resulting in a minimum SS of 80 mV/dec, a decent ON/OFF ratio (~10^4^), and a low gate leakage current (Supplementary Fig. 7a). To understand the sub-threshold behavior, local SS values are plotted in Fig. 2b after fitting of the experimental data to facilitate differentiation. The basic equation for SS can be expressed as:

1 $${\mathrm{SS}} = \eta \frac{{kT}}{q}{\mathrm{ln}}(10)$$

where *ɳ* is the ideality factor and can be expressed as:

2 $$\eta = 1 + \frac{{C_{{\mathrm{IT}}} + C_{\mathrm{D}}}}{{C_{{\mathrm{EDL}}}}}$$

*C*_IT_, *C*_D_, and *C*_EDL_ represent the interface trap capacitance, depletion capacitance, and effective electrolyte capacitance, respectively. Owing to the ultra-thin body of few-layer TMDs explored in this work, the channel can be assumed to be fully depleted (*C*_D_ ~0 F).

Using the minimum SS value of 80 mV/dec in Eq. (1), a value of *ɳ* = 1.33 is obtained for the CVD MoS_2_ FET, which leads to a minimum value of *C*_IT_ that is equal to 0.70 µF cm^−2^. The deviation of *ɳ* from ideal value (*ɳ* = 1) in transferred CVD MoS_2_ FETs can be attributed to the incorporation of impurities from transfer and fabrication process, as well as intrinsic crystal defects, which collectively lead to a finite interface trap capacitance (*C*_IT_)^40^.

Next, the field-effect mobility can be calculated using:

3 $${\mathrm{\mu }} = \frac{{\partial I_{{\mathrm{DS}}}}}{{\partial V_{{\mathrm{BG}}}}} \cdot \frac{L}{W} \cdot \frac{1}{{C_{{\mathrm{EDL}}}V_{{\mathrm{DS}}}}}$$

where *C*_EDL_, *L*, and *W* are the effective electrolyte capacitance, channel length, and width, respectively.

Using the maximum slope from the linear *I*_DS_*-V*_BG_ of Fig. 2a and a *C*_EDL_ value of ~2.10 µF cm^−2^, *µ*_e_ ~ 18 cm^2^V^−1^s^−1^ for the CVD MoS_2_ FET, which is consistent with the mobility values obtained for good-quality back-gated CVD MoS_2_ transistors^41,42^.

Next, we investigate the effect of voltage sweep rates on transfer characteristics. Sweep rates of 44 mV s^−1^, 9 mV s^−1^, and 1 mV s^−1^ are designated as fast, medium, and slow speeds, respectively. No significant variation is observed in transfer characteristics for these three different speeds, as shown in Fig. 2c, except for the hysteresis voltage, Δ*V*_TH_, which is 49 mV, 63 mV, and 112 6mV for slow, medium, and fast speeds, respectively (Supplementary Fig. 8). Notably, the Δ*V*_TH_ change between slow and medium sweep rates is negligible and of the order of thermal voltage (~ 25 mV). As a result, a medium sweep rate (9 mV s^−1^) is used throughout this work, unless otherwise stated. Output characteristics for the same device are shown in Fig. 2d. At small drain voltages, the output characteristics are linear, suggesting an Ohmic-like contact (Supplementary Fig. 7b). At high drain voltages (*V*_DS_ > *V*_BG_-*V*_TH_), current reaches saturation similar to a well behaved conventional FET^43^. Importantly, we note from the *I*_D_*-V*_D_ output characteristics of MoS_2_ FET, a crossover between channel pinch-off (*I*_D,sat_ *α* $V_{{\mathrm{OV}}}^2$) and velocity saturation (*I*_D,ʋsat_ *α V*_OV_*ʋ*_sat_) regime (see Supplementary Fig. 9 and Supplementary Note 2 for further clarification), at an overdrive voltage (*V*_OV_ = *V*_BG_ − *V*_TH_) of ~0.5 V (*V*_BG_ = 0.9 V, *V*_TH_ = 0.4 V), similar to the observation of a previous report^44^. Similar electrical characteristics (SS ~75 mV/dec, and ON/OFF ratio ~10^5^) are obtained for another CVD MoS_2_ FET (Supplementary Fig. 10).

With four-probe technique, the contact resistance (*R*_c_) for a single-layer CVD MoS_2_ FET has been determined to be ~40 kΩ.µm at *n* > 10^13^ cm^−2^ (Supplementary Fig. 11), which is of the same order of magnitude as the reported values (10–100 kΩ.µm) for a SiO_2_/Si back-gated FET with the same contact metal (Ni/1 L CVD MoS_2_) under a similar deposition condition (e-beam, ~10^−6^ Torr)^45^. Similar studies with ionic liquid top-gated devices have shown a significant reduction of contact resistance for both electrons and holes^1,46,47^. This, however, results in ambipolar transport. In this work, a desirable unipolar electron branch has been observed in Li-ion back-gated MoS_2_ FET (see Supplementary Note 3 for more details), which is favorable for realizing CMOS circuits.

To better understand the electrostatics of the Li-ion glass-based FET, schematics illustrating the chemical potentials of the Li-ion glass and species in contact (Ag and MoS_2_) with it are shown in Fig. 2e, f. In the OFF state, the spontaneous alignment of the Fermi levels (electrochemical potential, not shown here) is made by the diffusion of Li ions in the Li-ion glass into the inner region, leaving negatively charged vacancies (anions) behind (at the surfaces); therefore, the electrolyte may align its Fermi level to the Fermi levels of both materials in electrical contact with its surfaces by locally changing its composition and by the formation of EDLs to finally have $\bar \mu _{{\mathrm{Ag}}} - \bar \mu _{{\mathrm{MoS}}_2} = 0 = \mu _{{\mathrm{Ag}}} - \mu _{{\mathrm{MoS}}_2} + {\mathrm{e}}\Delta V_{{\mathrm{OCV}}}$, where $\bar \mu _{{\mathrm{Ag}}}$, $\bar \mu _{{\mathrm{MoS}}_2}$ are electrochemical potentials or Fermi levels, *μ*_Ag_, $\mu _{{\mathrm{MoS}}_2}$ are the chemical potentials and eΔ*V*_OCV_ is the open-circuit voltage in eV. The semiconductor and silver cannot change their chemical potentials without an exchange of electrons or holes, and therefore, in OFF state, the Fermi levels alignment is made by the electrolyte as shown in Fig. 2e. Once the back-gate voltage is applied to the silver end, the chemical potential of the silver decreases, whereas that of the MoS_2_ increases. MIM measurements in Fig. 5 show that the channel is formed at 0.30 V, which indicates that the Fermi level of the Li-ion glass is surpassed at 0.30 eV and the electrons at the surface of the MoS_2_ form an EDL with the Li-ion glass mobile cations to dynamically align their Fermi levels (Fig. 2f).

Further experiments are performed on MoS_2_, which is exfoliated directly onto Li-ion glass, in order to study the transport characteristics of multi-layer MoS_2_ FETs. The layers are identified by optical microscopy and confirmed using Raman spectroscopy (Supplementary Fig. 12). Transfer characteristics for mono-, bi-, tri-, and four-layer MoS_2_ FETs are shown in Supplementary Fig. 13, with device performance near 60 mV/dec. However, the limited device statistics preclude drawing conclusions on the thickness-dependent effect on the electrolytic substrate. The best field-effect device is obtained for 4 L MoS_2_ which demonstrates a SS of ~64 mV/dec, and an ON/OFF ratio above 10^6^, with a field-effect mobility approximately equal to ~20 cm^2^V^−1^s^−1^ (Supplementary Fig. 13).

### Transistor properties of p-type WSe_2_ FET

**Fig. 3:**
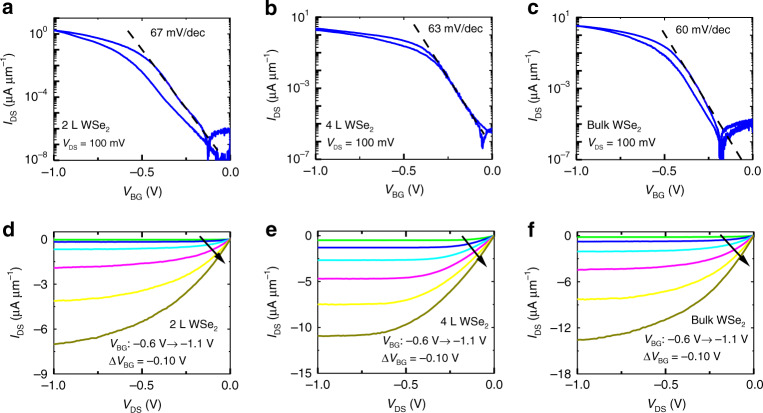
Electrical transport characteristics of exfoliated WSe_2_ on Li-ion glass. Transfer characteristics of **a** 2 L, **b** 4 L, and **c** bulk (~ 14 nm thick) WSe_2_ FETs all featuring *I*_ON_/*I*_OFF_ ~ 10^7^ and hysteresis voltage < 120 mV. |*V*_TH_ | is ~0.6 V, 0.34 V, and 0.5 V for 2 L, 4 L, and bulk, respectively. Output characteristics of **d** 2 L, **e** 4 L, and **f** bulk WSe_2_ FET. For small drain bias, a linear *I*_D_*-V*_D_ is observed indicating Ohmic-like contact. For higher drain bias, current saturation is observed.

To study the effect of EDL on p-type semiconductors, WSe_2_, a p-type 2D material, was exfoliated onto Li-ion glass substrate using commercial bulk WSe_2_ (from HQ Graphene). Transport characteristics for 2 L, 4 L, and bulk WSe_2_ FETs were investigated. The flakes are identified using optical microscopy and evaluated by Raman and PL characterization, which show good crystalline quality (Supplementary Fig. 14). Sub-threshold swings approaching the ideal limit of ~60 mV/dec were obtained from electrical transfer characteristics of WSe_2_ FETs as shown in Fig. 3. A field-effect mobility of ~25−40 cm^2^V^−1^s^−1^ has been calculated for the WSe_2_ FETs based on the capacitance of the EDL (*C*_EDL_). Negative threshold voltage and lack of electron transport indicates unipolar conduction with desirable e-mode transistor operation. The *I*_OFF_ is limited by the gate leakage current, similar to MoS_2_ FET (Supplementary Fig. 15a–c). A linear relationship at low *V*_DS_ (Supplementary Fig. 15d–f) and current saturation at higher *V*_DS_ (Fig. 3d–f) mostly caused by channel pinch-off similar to a well-behaved FET^43^, are observed (see Supplementary Fig. 9 and Supplementary Note 2 for further details).

Using the chemical potential of silver (−5.24 eV) as reference^48^, the Li-ion glass chemical potential, *μ*, is calculated to be *μ*(Li-ion glass) ≈ −5.07 eV, which makes *μ* (MoS_2_) = −5.37 eV and *μ*(WSe_2_) = −4.73 eV, *μ* (MoS_2_) − *μ*(WSe_2_) = −0.64 eV (Supplementary Fig. 16). Experimental results obtained in the literature show, *μ* (MoS_2_) = −4.77 ± 0.45 eV^48^ and *μ*(WSe_2_) = −4.61 ± 0.20 eV^49^, *μ* (MoS_2_) − *μ*(WSe_2_) = −0.16 eV. Surface phenomena such as the direction of the cut, crystalline disorder, polarization, and the number of atomic layers may influence the work functions; on the other hand, the Fermi levels are bulk dependent and, therefore, utilizing work functions are not always the best option to calculate the Fermi levels.

### CMOS inverter amplifier

**Fig. 4:**
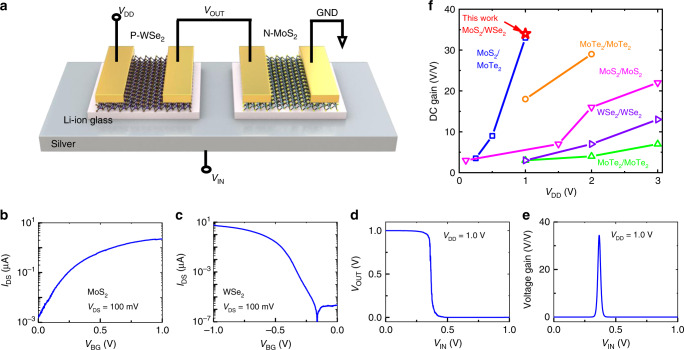
CMOS inverter based on n-type MoS_2_ and p-type WSe_2_ FETs. **a** Schematic of a CMOS inverter with the drain of p-WSe_2_ FET connected to the drain of n-MoS_2_ FET. Transfer characteristics of **b** n-type MoS_2_ FET. *V*_TH_ is ~0.25 V. **c** p-type WSe_2_ FET. *V*_TH_ is ~−0.50 V. **d** Output vs input voltage characteristics of the inverter. The mid-point voltage *V*_M_ is 0.38 V. Noise margin high (NM_H_) and low (NM_L_) as calculated from the graph are 0.56 V and 0.27 V, respectively. **e** Voltage gain vs. input voltage curve with a maximum gain of ~34 at *V*_IN_ = 0.37 V. **f** DC gain vs. supply voltage (*V*_DD_) of our n-MoS_2_/p-WSe_2_ CMOS inverter (red) along with other CMOS inverters from the literature such as n-MoS_2_/p-MoTe_2_ (blue)^60^, n-MoTe_2_/ p-MoTe_2_ (orange)^61^, n-MoS_2_/p-MoS_2_ (magenta)^62^, n-WSe_2_/ p-WSe_2_ (violet)^63^, and n-MoTe_2_/ p-MoTe_2_ (green)^64^.

With suitable electrostatic and transport characteristics in both n-type MoS_2_ and p-type WSe_2_ FETs, a CMOS inverter was realized by connecting these two types of FETs. Figure 4a–c show a schematic diagram of the CMOS inverter with the biasing scheme and the associated transfer characteristics. Voltage transfer characteristics (VTC) of the CMOS inverter are shown in Fig. 4d. The VTC has a full logic swing with a 1 V supply, and offers a steep transition between the two logic states (LOW and HIGH). The mid-point (where *V*_IN_ = *V*_OUT_) or the switching threshold voltage *V*_M_ = 0.38 V is slightly less than the ideal value of *V*_DD_/2 = 0.5 V. Input/output high/low voltages are found from the VTC curve where the slope = −1. The values are as follows: *V*_IH_ = 0.40 V, *V*_IL_ = 0.33 V, *V*_OH_ = 0.96 V, and *V*_OL_ = 0.06 V. Noise margin high/low are calculated to be NM_H_ (*V*_OH_ − *V*_IH_) = 0.56 V and NM_L_ (*V*_IL_ − *V*_OL_) = 0.27 V at a supply voltage of *V*_DD_ = 1 V. By normalizing the values with respect to supply voltage (*V*_DD_ = 1 V), we get a noise margin of 56% (NM_H_) and 27% (NM_L_) from high/low to low/high transitions, respectively. This means that 56% (27%) noise can be tolerated in the process of high (low) to low (high) logic conversion, with the state detectable without error. By using different channel widths, an identical ON-current can be achieved for both n-type and p-type FETs, which may facilitate a symmetric VTC and better noise margins.

Another important figure of merit for CMOS inverters is the DC voltage gain (Fig. 4e), which is calculated from the slope (d*V*_OUT_/d*V*_IN_). A maximum gain of ~34 is obtained at an input voltage of *V*_IN_ = 0.37 V. For comparison, the DC gain vs. supply voltage characteristics of other TMD-based realistic CMOS inverters are plotted together in Fig. 4f. We note that the DC gain in this work, obtained at a supply voltage of 1 V, is higher than the other reported values of practical solid-state CMOS inverters. Detailed information (such as supporting substrate, gate dielectric, input voltage range) of other reported works are summarized in Supplementary Table 1. The output current and static DC power (Power = *V*_DD_ × *I*_OUT_) are determined to be ~0.30 µA and ~300 nW, respectively (Supplementary Fig. 17).

### Microwave impedance microscopy (MIM)

**Fig. 5:**
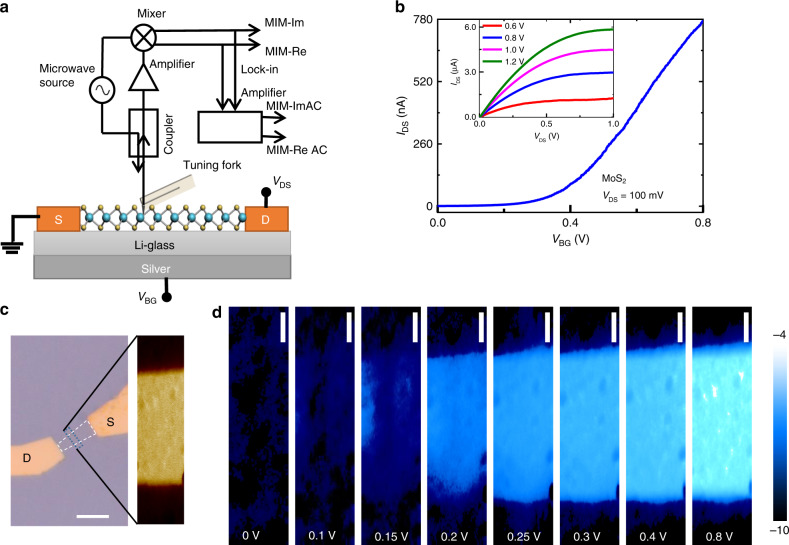
MIM measurement of MoS_2_ FET. **a** Schematic diagram of the device and the tuning-fork-based MIM setup. The 1-GHz microwave signal is guided to the tip through an impedance match section, and the reflected signal is detected by the MIM electronics. The carrier density is tuned by the back-gate voltage *V*_BG_. **b** Transfer characteristics of the MoS_2_ FET (*L* = 6 µm) for *V*_DS_ = 100 mV. Inset: Output characteristics for gate voltages from 0.60 V to 1.20 V in steps of 200 mV. **c** Optical image of the device, where white dashed lines show the channel boundary and the blue dotted line shows the section of the channel where MIM is performed. Inset shows MIM map of the selected channel region. Scale bar is 5 µm. **d** Sheet conductance map in a section of the FET channel at different gate voltages. The color scale represents the common logarithm of sheet conductance in S/◻. All scale bars are 500 nm.

The transistor-based measurements discussed above provide information on the global transport behavior over the entire channel area. In order to gain more insight on the gate-dependent local conductance, we have performed tuning-fork (TF) based microwave impedance microscopy (MIM)^50^, as schematically illustrated in Fig. 5a. An electrochemically etched tungsten tip (~120 nm) is attached to a quartz TF to provide the topographic feedback. The 1 GHz microwave signal is delivered to the tip and the reflected signal is detected by the MIM electronics. The distance modulation leads to the periodic change of MIM signals at the TF frequency, which are demodulated by a lock-in amplifier to form AC_MIM images (Methods and Supplementary Fig. 18). Quantification of the AC_MIM signals into local sheet conductance using finite-element analysis (FEA) can be found in Supplementary Fig. 18. The transfer characteristics of a long-channel MoS_2_ FET (*L* = 6 µm) at *V*_DS_ = 100 mV are shown in Fig. 5b. Figure 5d shows the gate-dependent channel conductance maps in a section of the same device (Fig. 5c). The insulator-to-metal transition can be clearly observed from the images. For *V*_BG_ below 0.1 V, there is little contrast between the MoS_2_ region and the substrate, indicating that the channel is highly resistive. As *V*_BG_ gradually increases to 0.15 V, charge carriers start to appear near the two electrodes, similar to the behavior observed in the ion gel-gated ZnO FET^51^. The conductive regions continue to extend towards the center at *V*_BG_ = 0.20 V. Finally, as the FET is turned on beyond *V*_BG_ = 0.3 V, the MoS_2_ channel is uniformly conductive within the spatial resolution of the MIM (~100 nm). Interestingly, the behavior is in sharp contrast to that in a previously reported MoS_2_ FET fabricated on conventional SiO_2_/Si substrate and capped by an Al_2_O_3_ layer, where strong mesoscopic inhomogeneity was observed^26^. The result may be indicative of a smooth interface between the TMD and electrolytic substrate. The homogeneous channel formation may also be attributed to the suppression of charged impurity scattering effect (usually distributed non-uniformly over the channel) by increased and efficient dielectric screening of the underlying high-ҡ dielectric (*ҡ* = 35 for Li-ion glass)^52^ substrate^53,54^. Under these circumstances, surface polar phonons of the underlying substrate (Li-ion glass) possibly limit the electron transport (and mean free path) in MoS_2_ on Li-ion solid electrolytic substrate^55–57^.

## Discussion

In summary, we have presented a lithium-ion based solid electrolyte as a promising platform substrate for transistor device studies. In addition to offering significant advantages over the ionic liquid gating technique, this substrate can be readily exploited as a back-gate with ideal gate control. As an exemplary nanomaterial, 2D transition metal dichalcogenide semiconductor transistors afford sub-threshold control approaching the ideal limit of 60 mV/dec, high ON/OFF ratios above 10^6^, and relatively high-mobility within the range of 18−40 cm^2^V^−1^s^−1^. Remarkably these performance metrics are achieved with a 1 V voltage supply for relatively long (micro-meter) channel lengths, indicating promising prospects for devices with smaller channel dimensions particularly with regards to power supply reduction under field-invariant scaling theory^58^.

In addition, a CMOS inverter amplifier has been realized using n-type MoS_2_ and p-type WSe_2_, which individually display the desirable unipolar characteristics. The CMOS amplifier boasts a voltage gain of ~ 34, which is the highest reported for similar amplifiers with low voltage supply (≤1 V). These features of the Li-ion glass, together with the formation of a uniform conduction channel in the TMD upon gating, as evidenced by MIM measurement, make this substrate an attractive choice for advanced thin-film devices and associated device physics.

## Methods

### Substrate preparation

Li-ion glass substrate is composed of Li_2_O-Al_2_O_3_-SiO_2_-P_2_O_5_-TiO_2_-GeO_2_ and came in the form of polished plates named as Lithium-Ion Conductive Glass Ceramic (LICGC^TM^) AG-01 from Ohara Corporation. We purchased polished square plates (25.4 mm by 25.4 mm and 150 µm thick) of AG-01 LICGC and patterned alignment marks on them using photolithography and a subsequent e-beam metal (20 nm/30 nm Ni/Au) evaporation step. The samples with alignment marks are then cut into standard sizes (6.3 mm × 6.3 mm) with a dicing saw (ADT 7100 Series System) using a resin blade (CA-010-325-100-H). Back-side of the electrolyte substrate is silver-coated with a Q-tip for back-gate measurement.

### 2D materials preparation

Bulk MoS_2_ (natural) and WSe_2_ (synthetic) crystals are purchased from commercial vendors 2D Semiconductors and HQ Graphene, respectively. MoS_2_ and WSe_2_ is exfoliated from bulk crystal using ultra tape (Ultra Tape 1310) and transferred onto Li-ion glass substrates from ultra-tape using polydimethylsiloxane (PDMS) stamp. A subsequent annealing step is done in high vacuum (10^−7^ Torr) at 340 °C for ~8 h to remove tape/organic residues introduced during exfoliation/transfer process. CVD MoS_2_ is grown on a pre-cleaned highly doped SiO_2_/Si substrate in a single zone furnace at 850 °C using molybdenum oxide (MoO_3_) and sulfur (S) powders as precursors. The CVD grown material is then transferred on Li-ion glass substrate by poly (methyl methacrylate) (PMMA)-assisted wet transfer using sodium hydroxide (NaOH) of 6 M (6 mols of NaOH in 1 liter of H_2_O) as etchant.

### 2D materials characterization

Optical characterization was done using Olympus microscope (BX53M) and their proprietary software Stream Essentials. Since the contrast of the flakes on glass is not good in bright field, we use dark-field mode to see and capture the images. Raman and PL spectroscopy are performed in a Renishaw inVia micro-Raman system. Excitation wavelength of 532 nm with an incident beam power of ~1 mW and exposure time of 10 s is used for Raman. A 3000 l/mm grating is used for < 5 cm^−1^ resolution. For photoluminescence spectroscopy, excitation wavelength of 532 nm with incident power <1 mW and exposure time ~10 s is used. A 1200 l/mm grating is used for PL measurements.

### Device fabrication

EBL is used to pattern contact and measurement pads. E-beam metal evaporation is then used to deposit contact metals. Ni/Au (20 nm/30 nm) and Pd/Au (5 nm/5 nm) are used for MoS_2_ and WSe_2_ contact metals, respectively. The channel region is defined with an EBL and a subsequent plasma etching step. CF_4_/O_2_ and Cl_2_/O_2_ plasmas are used to etch WSe_2_ and MoS_2_, respectively. In some of the EBLs, we employ an e-spacer charge compensation layer, but no significant difference is observed with/without this step.

### Electrical characterization

All electrical DC measurements are performed on a Cascade Microtech Summit 11000B-AP probe station using an Agilent 4156C parameter analyzer in ambient at room temperature. Quasi-static CV is measured with Keysight B1500 parameter analyzer. For frequency-dependent capacitance, HIOKI 3536 LCR meter has been used.

### TF-MIM

A tuning-fork-based MIM is employed to map the local conductivity. The technique utilizes a tuning-fork-based AFM combined with a 1 GHz microwave signal guided by an electrochemically etched tungsten tip (25 µm diameter wire), which is glued on the tuning fork (resonant frequency ~38 kHz). A Zurich HF2LI lock-in amplifier is used to drive the tuning-fork tip at its resonant frequency in the driving amplitude modulation (DAM) mode^50^. The topography feedback is realized by a commercial AFM system (Park XE-70). The AC_MIM signals are demodulated by an SR830 lock-in amplifier and then acquired by the Park system. During the measurements, source and drain electrodes are grounded, DC offset of the tip is set to zero through a bias-tee, and the back-gate voltage is applied using Keithley 2400 Source Measurement Unit (SMU) to modulate the carrier density.

### Finite-element analysis (FEA)

Finite-element analysis is performed by COMSOL 4.4 to verify the response of AC_MIM signals on MoS_2_ devices. Since the lateral dimensions of flakes are much larger than the MIM tip diameter (120 nm), the 2D axisymmetric model can be used here. The device and substrate are characterized by the following parameters: MoS_2_: *t* (thickness) = 3 nm, *w* (width) = 6 µm and *ε* (dielectric constant) = 7^59^. Li-ion glass substrate: *t* = 150 µm, *ε* = 35^52^. We followed the standard procedure described in ref. ^50^ to convert the demodulated tip−sample admittance to the AC_MIM output based on the calibration of our electronics. The tip taps at 0.5 nm height above the sample surface with an amplitude of 14 nm and a frequency of 38 kHz.

## Supplementary information

Supplementary Information

## Data Availability

The data that support the plots within this paper and other findings of this study are available from corresponding author upon reasonable request.
