# Author Correction: Lithium-ion electrolytic substrates for sub-1V high-performance transition metal dichalcogenide transistors and amplifiers

**DOI:** 10.1038/s41467-021-23513-1

**Published:** 2021-05-12

**Authors:** Md Hasibul Alam, Zifan Xu, Sayema Chowdhury, Zhanzhi Jiang, Deepyanti Taneja, Sanjay K. Banerjee, Keji Lai, Maria Helena Braga, Deji Akinwande

**Affiliations:** 1 Microelectronics Research Center, Department of Electrical and Computer Engineering, The University of Texas, Austin, TX 78758 USA; 2 Department of Physics, The University of Texas, Austin, TX 78712 USA; 3 LAETA, Engineering Physics Department, Engineering Faculty, University of Porto, R. Dr. Roberto Frias s/n, 4200-465 Porto, Portugal

**Keywords:** Engineering, Materials science, Nanoscience and technology, Physics

Correction to: *Nature Communications* 10.1038/s41467-020-17006-w, published online 24 June 2020.

The original version of this Article misspelled the following number in the Acknowledgements: ‘DMR-1720595’, which incorrectly read ‘DMR-172059’.

This has now been corrected in both the PDF and HTML versions of the Article.

