# Supplementary material for: Lithium-ion electrolytic substrates for sub-1V high-performance transition metal dichalcogenide transistors and amplifiers
Source: Nat Commun. 2020 Jun 24;11:3203. doi: 10.1038/s41467-020-17006-w (PMC7314772; doi:10.1038/s41467-020-17006-w)
Supplement: Supplementary file 1 — Supplementary Information [file 41467_2020_17006_MOESM1_ESM.pdf]

Lithium-ion electrolytic substrates for sub-1V high-performance transition metal dichalcogenide transistors and amplifiers

Supporting Information

Md Hasibul Alam[1], Zifan Xu[2], Sayema Chowdhury[1], Zhanzhi Jiang[2], Deepyanti Taneja[1], Sanjay K. Banerjee[1], Keji Lai[2], Maria Helena Braga[3] and Deji Akinwande[*,1]

[1]Microelectronics Research Center, Department of Electrical and Computer Engineering, The University of Texas, Austin, Texas 78758, United States

[2]Department of Physics, The University of Texas, Austin, Texas 78712, United States

[3]LAETA, Engineering Physics Department, Engineering Faculty, University of Porto, R. Dr. Roberto Frias s/n, 4200-465 Porto, Portugal

Supplementary Figures

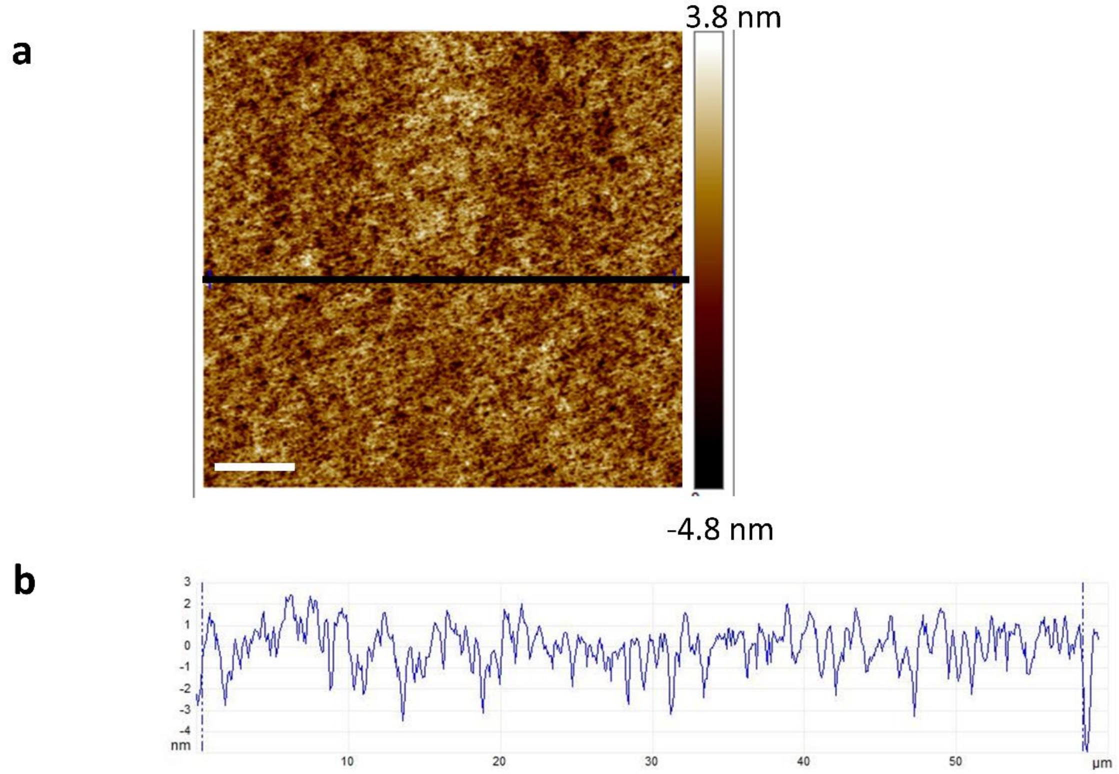

Supplementary Figure 1. AFM image of Li-ion glass substrate surface. (a) A scan of 60 µm by 60 µm area randomly selected to obtain substrate roughness. The average and RMS roughness are ~ 0.93 nm and ~1.19 nm, respectively. Scale bar is 10 µm. (b) The line scan for roughness along the black line in (a).

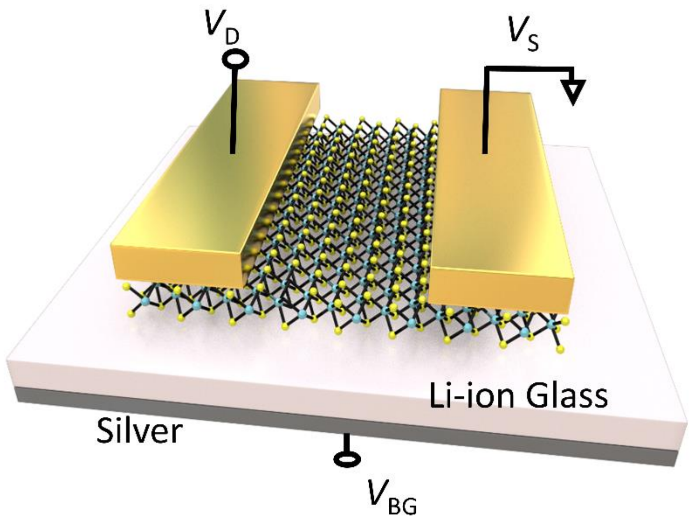

Supplementary Figure 2. Schematic of TMD back-gate transistor. Li-ion solid electrolyte works as back-gate dielectric/substrate and silver paint as a back-gate metal. The bias at the back-gate, drain and source is labeled as V_{BG}, V_D and V_S, respectively. Source is grounded whereas DC voltage is applied to the drain and gate terminals. Ni (Pd) is used as source/drain contact metals for MoS_2 (WSe_2) unless otherwise stated.

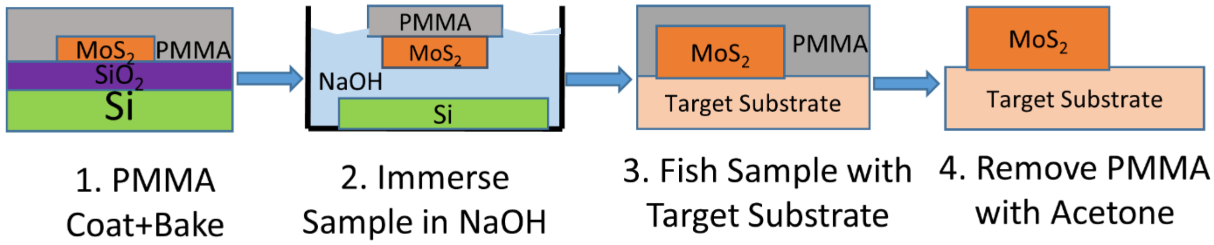

Supplementary Figure 3. Schematic of wet transfer method (not drawn to scale). The process involves i) delamination of MoS_2 from growth substrate using NaOH etchant, ii) fishing it onto target (Li-ion glass) substrate, iii) removal of PMMA and finally iv) annealing the sample.

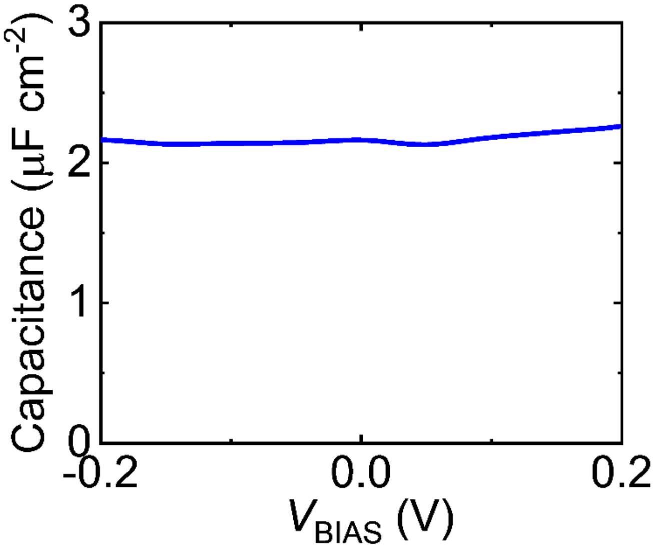

Supplementary Figure 4. Quasi-static capacitance voltage characteristics of a Li-ion glass substrate. Ni (20 nm) is used as both top and bottom electrode. The data is obtained by using B1500 semiconductor parameter analyzer. We note an average value of capacitance is equal to 2.15 μF cm^{-2}.

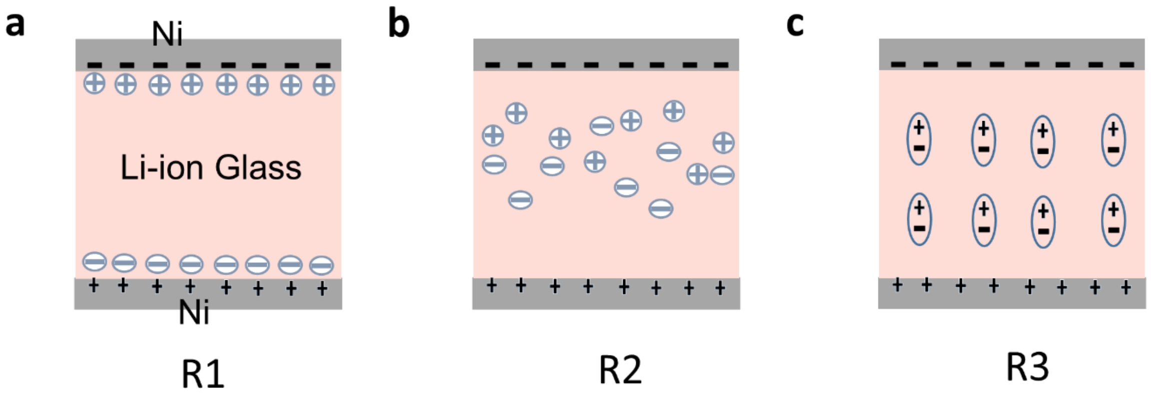

Supplementary Figure 5. Various regions of operation of Li-ion solid electrolyte. The frequency spectrum can be divided into 3 distinct regions: i) R1 where EDL is formed, ii) R2 where ion migration dominates, and iii) R3 where the bulk Li-ion glass works as a dielectric. Schematic diagram of (a) R1, (b) R2, and (c) R3 region. (a) R1 is in the frequency range <1.3 kHz where EDL is formed and the phase angle is close to -83˚. (b) R2 ranges from ~1.3 kHz to ~89 kHz where ion migration dominates. (c) R3 frequencies are above 89 kHz where the bulk Li-ion glass works as a dielectric.

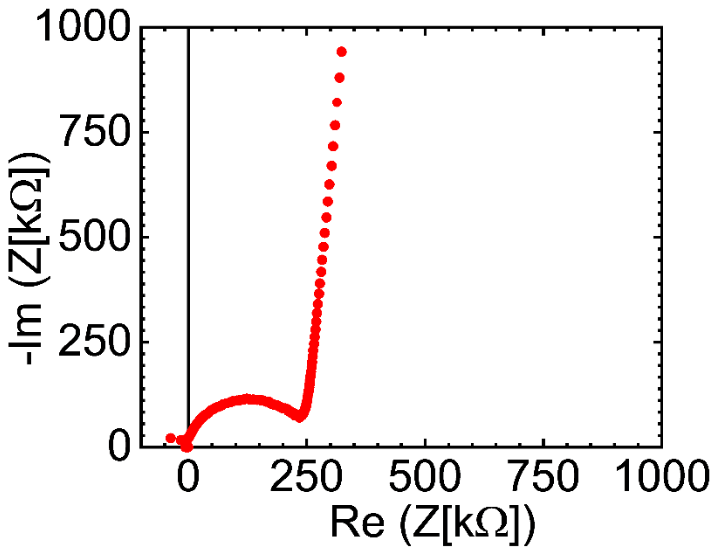

Supplementary Figure 6. Nyquist plot calculated from the data of experiment in Fig. 1f. The resistance, and consequently the conductivity, was calculated from Re (Z [Ω]) in the semi-circle (R= 2.49 × 10^5 Ω) using $\sigma = \frac{1}{R}\frac{d}{A}$, where A=165 μm ×165 μm is the area of electrode and d= 150 μm is the distance between the two electrodes.

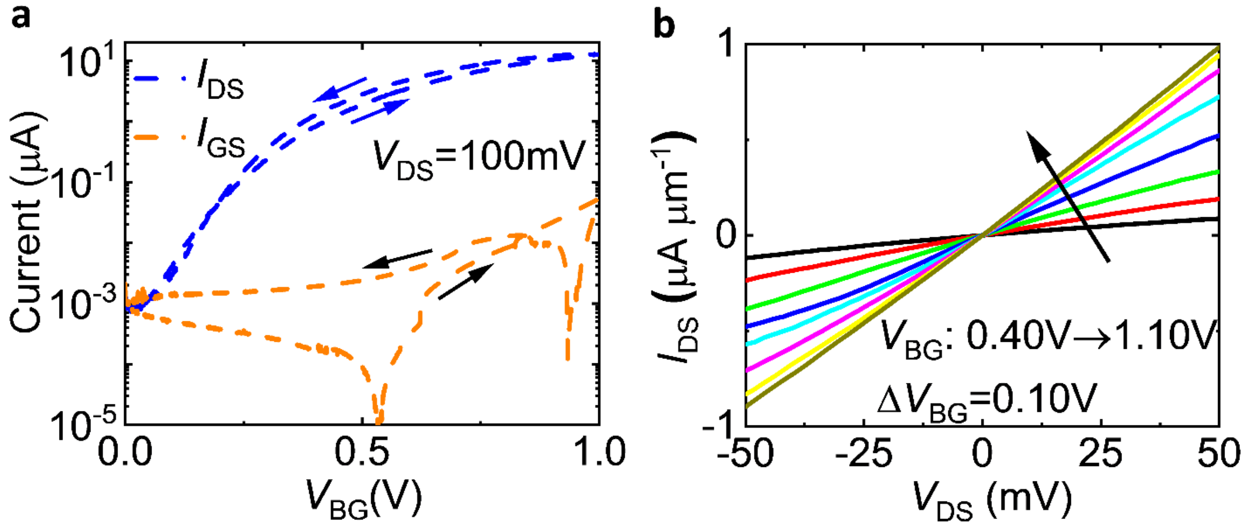

Supplementary Figure 7. Electrical transport characteristics of a CVD MoS_2 FET. (a) Gate leakage current vs. back gate (V_{BG}) voltage for monolayer CVD MoS_2 FET (L=1 μm, W=5 μm). (b) Output characteristics for small V_{DS} at different back-gate voltages for the same FET.

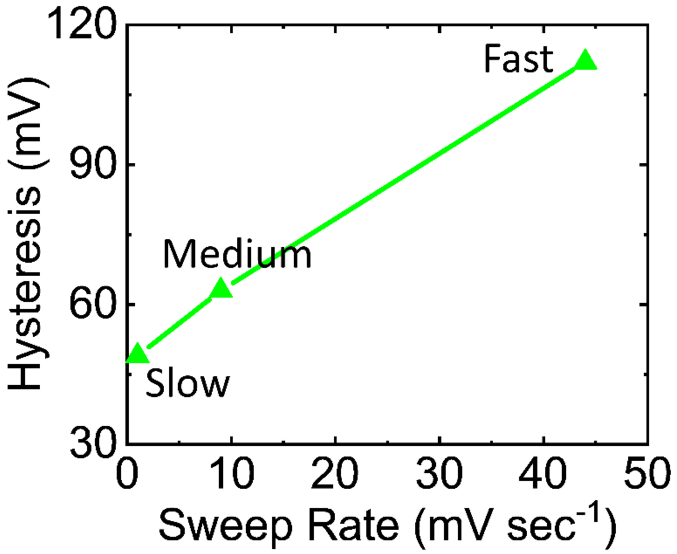

Supplementary Figure 8. Hysteresis voltage vs. sweep rate. In this work, fast, medium and slow speeds correspond to the sweep rates of 44 mV sec^{-1}, 9 mV sec^{-1} and 1 mV sec^{-1}, respectively.

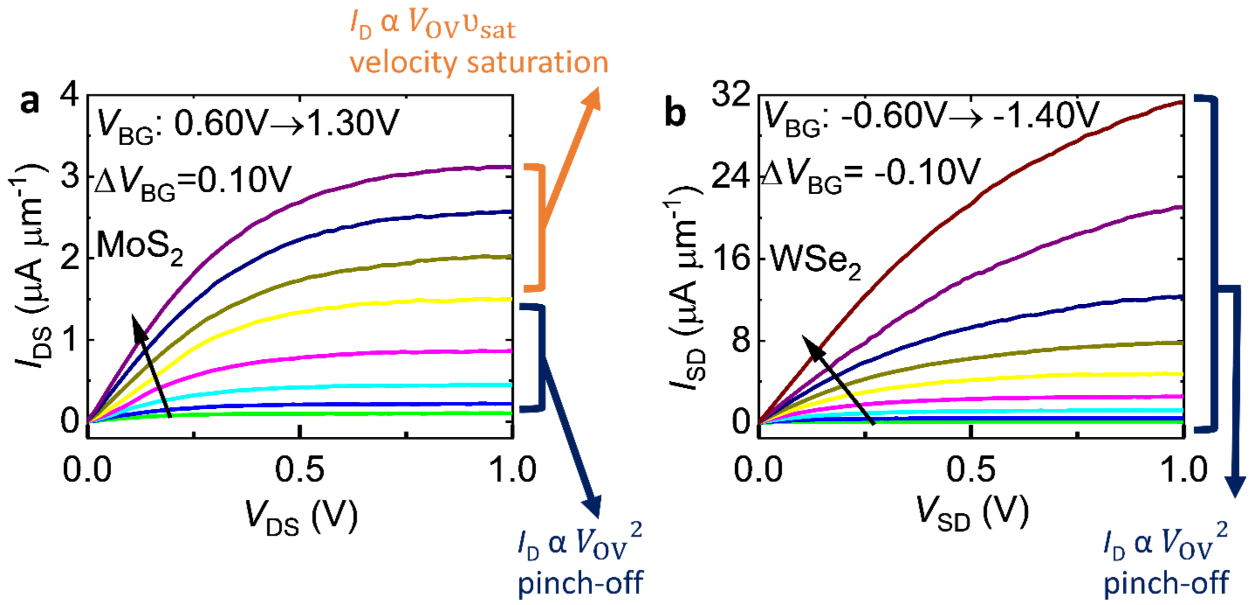

Supplementary Figure 9. Drain current saturation in TMDs with pinch-off/velocity saturation. (a) I_D-V_D characteristics of a MoS_2 FET. The saturation current is due to pinch-off up to a back-gate voltage of 1.00 V and after that it can be attributed to velocity saturation. Saturation current follows quadratic ($I_{D,sat}$ α V_{OV}^2) and linear ($I_{D,υsat}$ α $V_{OV}υ_{sat}$) relationship with gate overdrive voltage (V_{OV}=V_{BG}-V_{TH}) for pinch-off and velocity saturation, respectively. (b) I_D-V_D characteristics of a WSe_2 FET. The saturation is due to pinch-off in the measured gate voltage range (V_{BG} up to -1.4 V). The absence of velocity saturation may be attributed to a higher critical field for WSe_2 FET on Li-ion glass substrate.

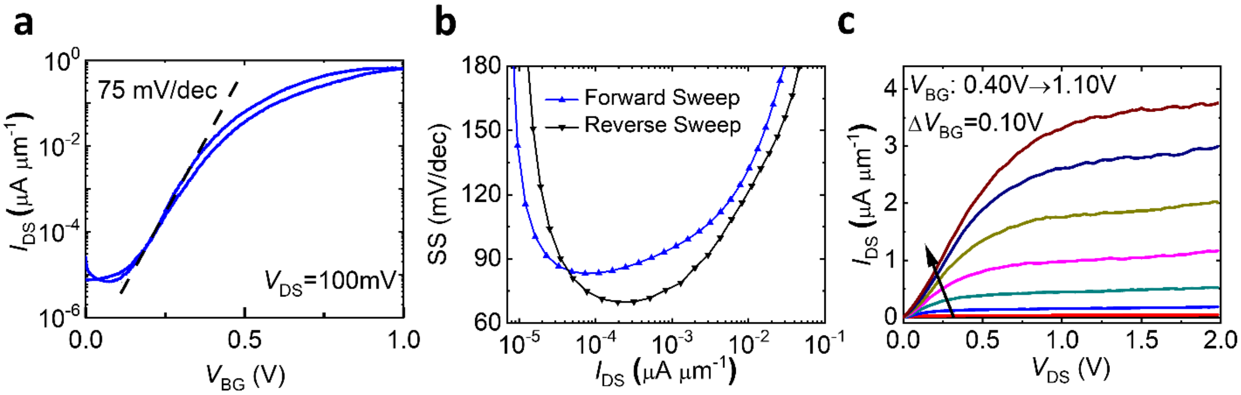

Supplementary Figure 10. Electrical transport characteristics of monolayer CVD MoS_2 FET (L=1.2μm, W=5 μm). (a) Transfer characteristics at V_{DS}=100mV. (b) SS vs. $I_{DS.}$ SS_{min} are 83 mV/dec and 70 mV/dec for FW and BW sweeps respectively. (c) Output characteristics for different gate voltages.

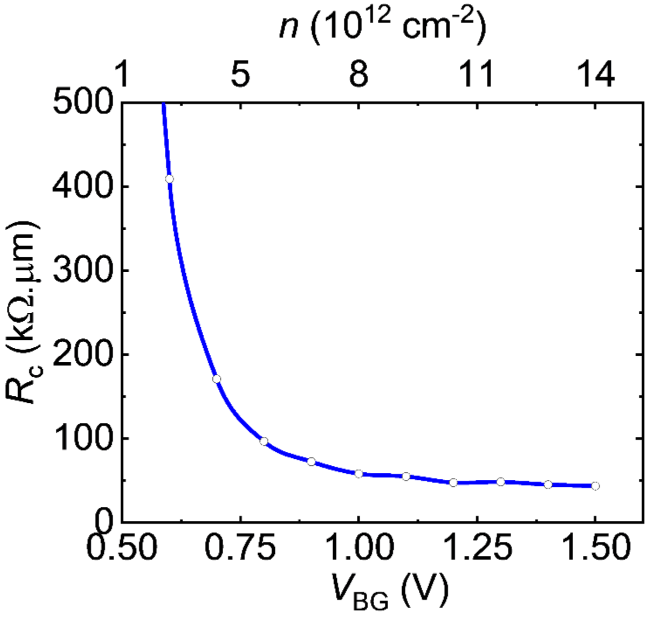

Supplementary Figure 11. Measured R_c vs. n (V_{BG}) for a single layer CVD MoS_2 transferred on Li-ion glass. R_c reaches ~ 40 kΩ.μm for $n > 10^{13}$ cm^{-2} or $V_{BG} > 1.25$ V, extracted using four probe technique. The contact metal is Ni.

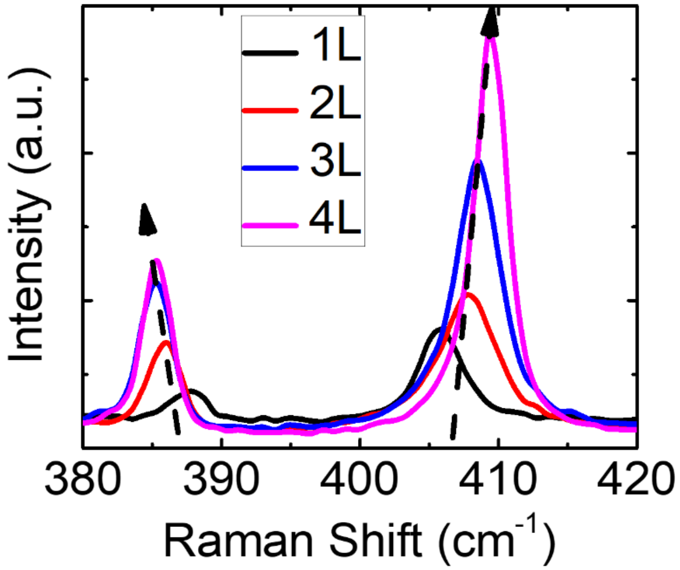

Supplementary Figure 12. Raman spectrum for 1L, 2L, 3L and 4L exfoliated MoS_2. Peak separation (distance between A_{1g} and E^1_{2g} peaks) agrees well with the reported value in literature for various layer thick MoS_2.[1]

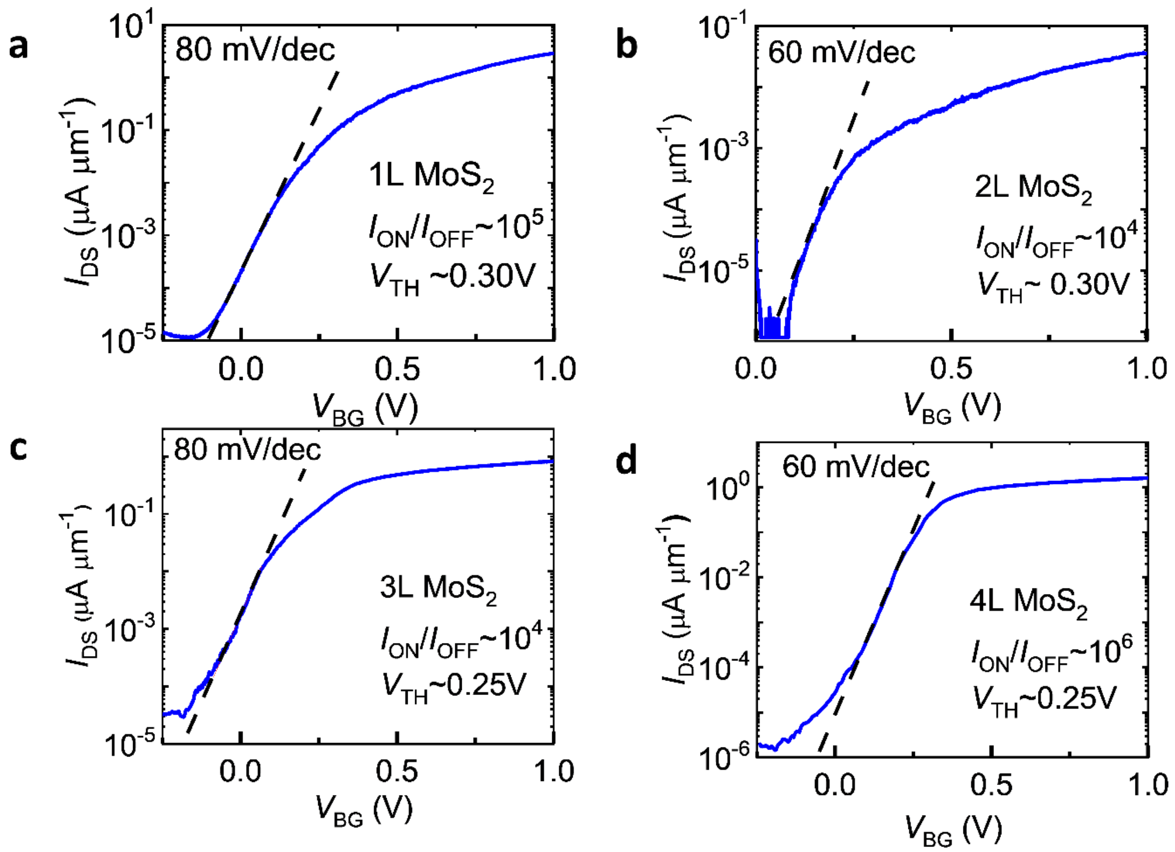

Supplementary Figure 13. Transfer characteristics of a (a) 1L, (b) 2L, (c) 3L and (d) 4L exfoliated MoS_2 FET. V_{TH}, ON/OFF ratios and sub-threshold swings are in the range of 0.25 V- 0.30 V, 10^4- 10^6, and 60-80 mV/dec, respectively for various layer thick MoS_2 FETs.

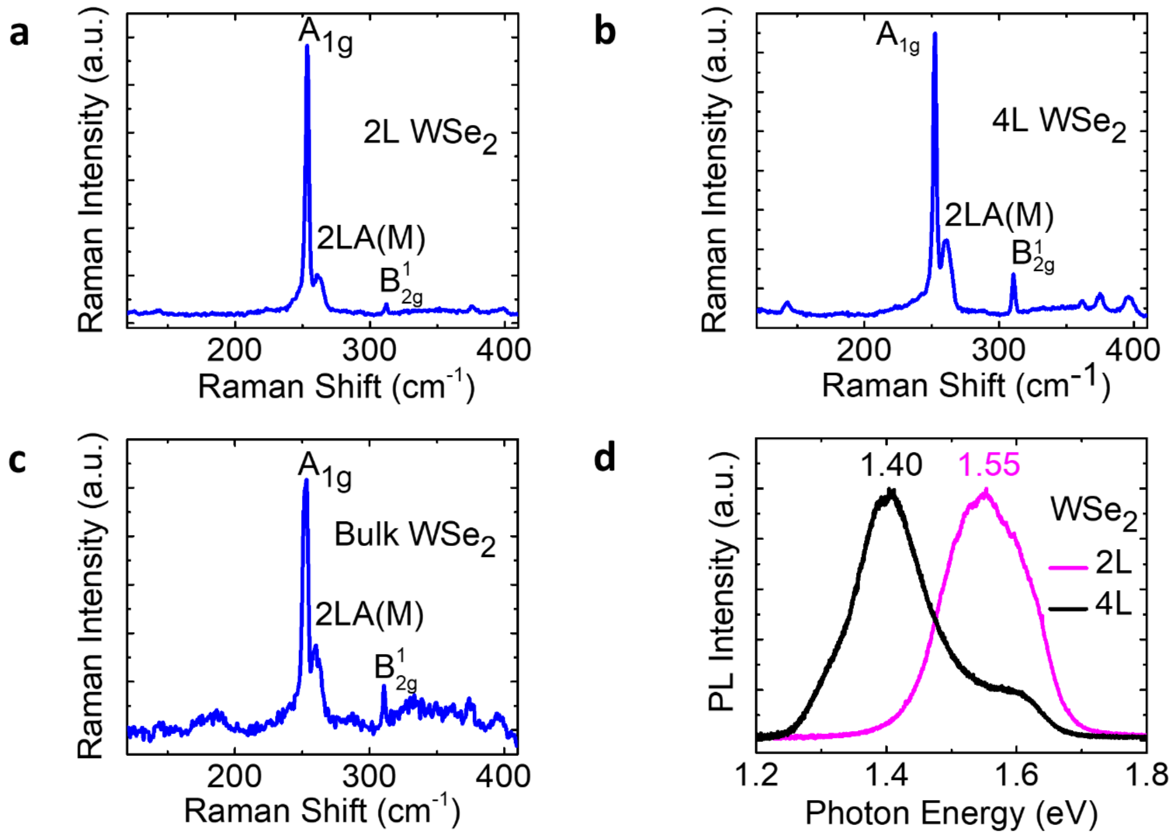

Supplementary Figure 14. Raman/PL spectrums for various layer WSe_2. Raman spectrum of exfoliated (a) 2L flake. Positions of A_{1g}, 2LA (M) and B^1_{2g} modes are 253.5 cm^{-1}, 261.3 cm^{-1} and 312.6 cm^{-1}, respectively. (b) 4L flake. Positions of A_{1g}, 2LA (M) and B^1_{2g} modes are 252.6 cm^{-1}, 260.4 cm^{-1} and 310.7 cm^{-1}, respectively. (c) Bulk flake (~14 nm). Positions of A_{1g}, 2LA (M) and B^1_{2g} modes are 253.1 cm^{-1}, 260.4 cm^{-1} and 310.3 cm^{-1}, respectively. (d) Photoluminescence spectrum of the 2L and 4L WSe_2 flake. FWHM of 2L and 4L are 152 meV and 102 meV, respectively.

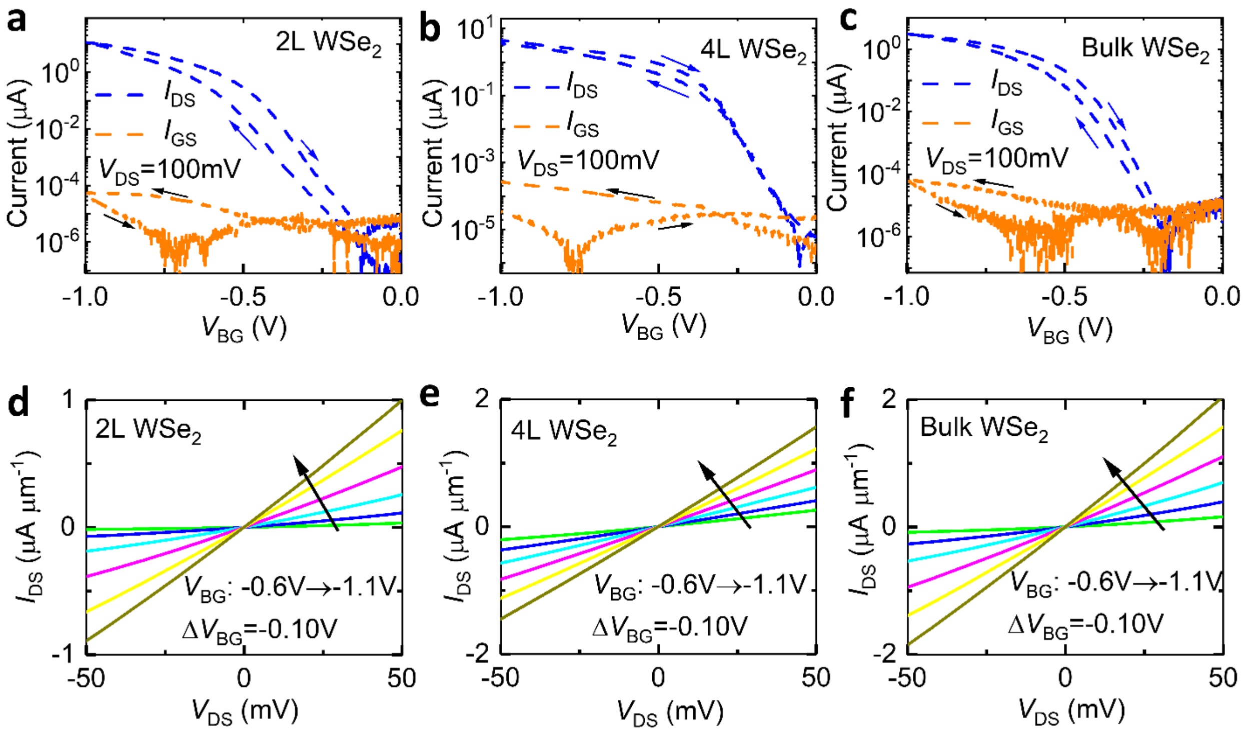

Supplementary Figure 15. Gate leakage current and linear I_D-V_D for various layer thick WSe_2. Drain current of (a) 2L, (b) 4L and (c) bulk WSe_2 FET. I_{DS}-V_{DS} relationship at small V_{DS} for a (d) 2L, (e) 4L and (f) bulk WSe_2 FET.

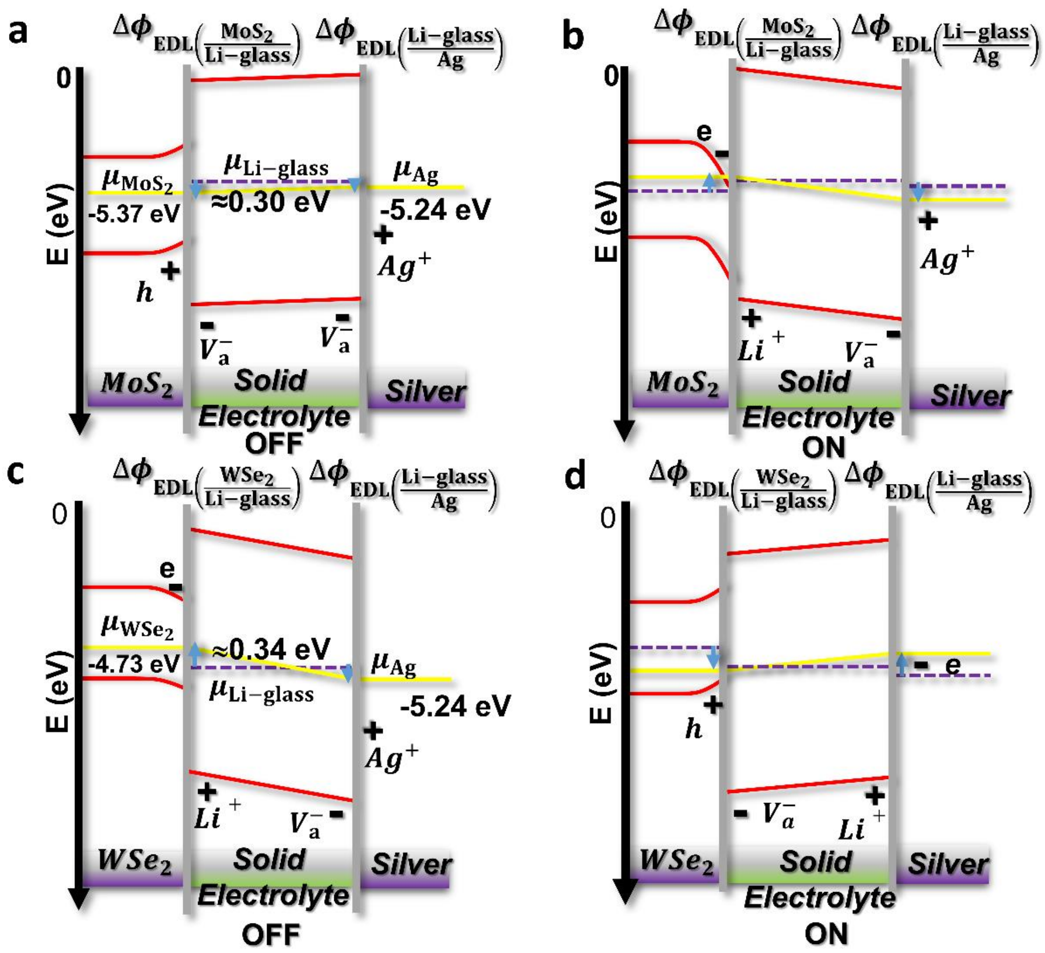

Supplementary Figure 16. Schematics illustrating the chemical potentials in ON and OFF state for a TMD FET. The species in electrical contact within the FET back-gate to drain align their Fermi levels (electrochemical potential, not shown here) by the formation of EDLs which are represented with the chemical potentials (Fermi levels of the electrically insulated species). The purple dashed line and solid yellow line represents the initial and final state of the chemical potentials, respectively. (a) MoS_2 FET in the OFF state, the Fermi level (electrochemical potential, not shown here) alignment is made spontaneously; the Li ions in Li-ion glass diffuse to the inner region leaving negatively charged vacancies behind (at the surfaces) which with the positively charged vacancies/cations constitute EDLs with the species in contact with the Li-ion glass (MoS_2 and Ag). (b) MoS_2 FET in the ON state after channel formation; the V_{BG} to form the channel at the surface with the Li-ion glass (0.30 V) is directly obtained by MIM measurements of MoS_2 FET (Fig. 5). (c) WSe_2 FET in OFF mode, the alignment of the Fermi levels is made spontaneously; the Li-ions diffuse to the surface of the Li-ion glass layer in electrical contact with WSe_2 leaving negatively charged vacancies behind (at the surface with Ag) constituting EDLs at both interfaces to allow Fermi levels alignment. (d) WSe_2 FET in ON mode after channel formation, V_{BG} to form the channel (0.34 V) was obtained indirectly in I_{DS} - V_{BG} measurements in Fig. 3b, when the WSe_2 aligns its Fermi level with the Li-ion glass. The dynamic alignment between the Fermi level of Ag and Li-ion glass was obtained at approximately -0.17 eV (Fig. 3b) when the drain current is essentially negligible (no EDL at the interface of Li-ion glass/Ag).[2]

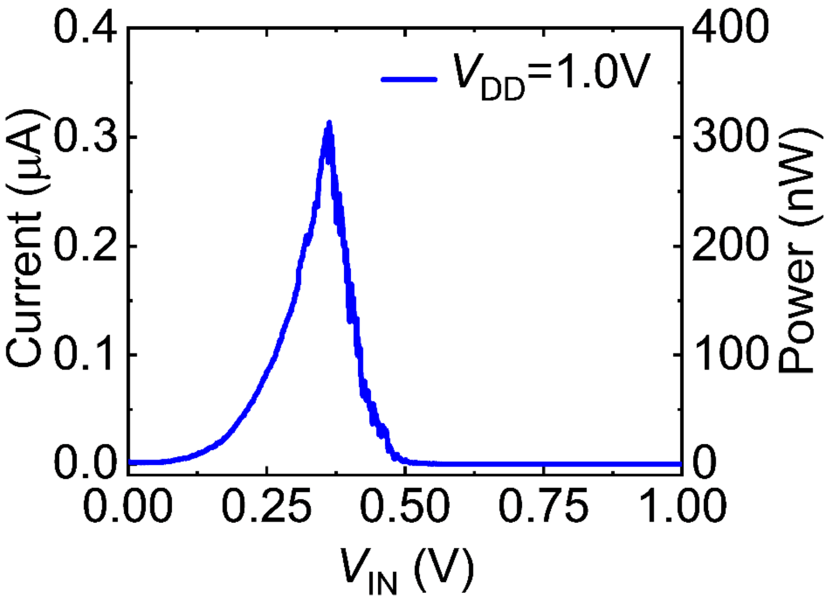

Supplementary Figure 17. Output current (static power) vs. input voltage of the inverter. Static power (Power=V_{DD}×I_{OUT}) is shown on the right axis of the same graph.

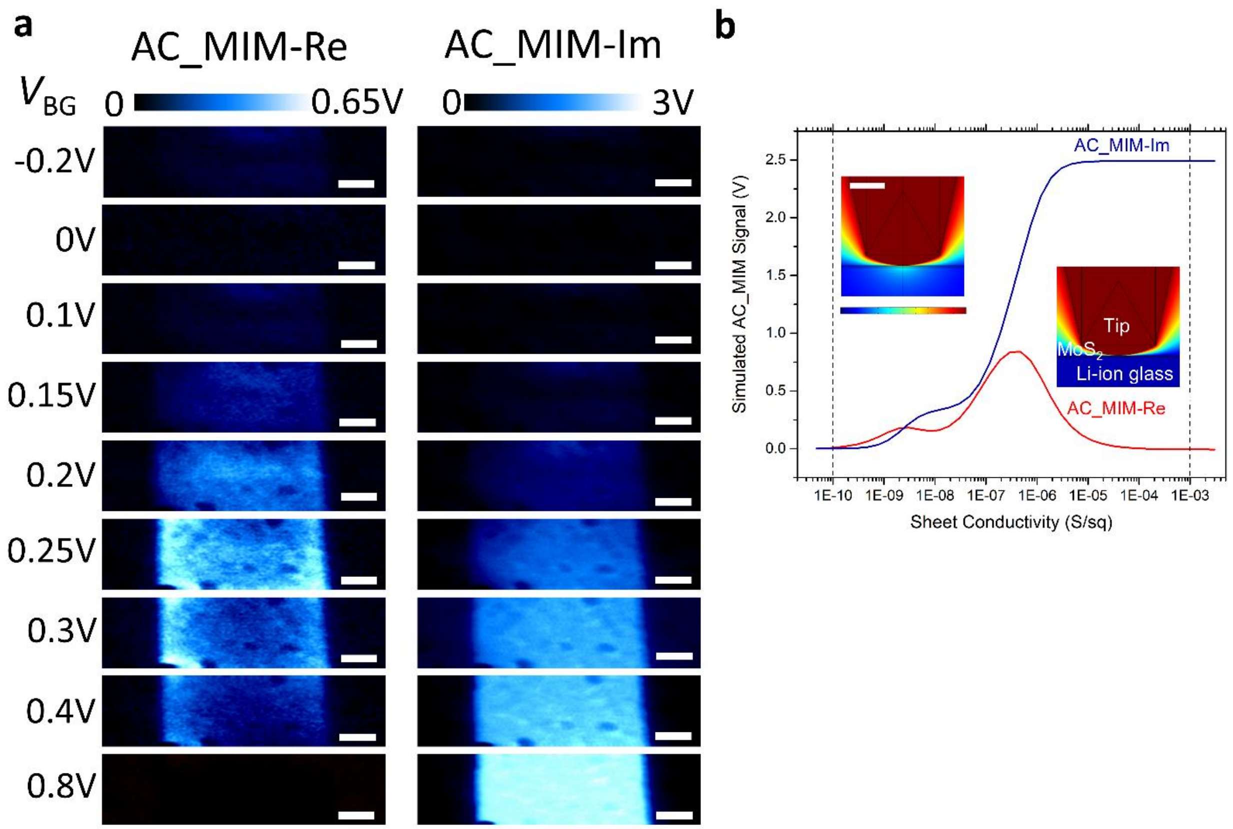

Supplementary Figure 18. MIM data of a MoS_2 FET on Li-ion substrate. (a) MIM maps of the selected channel region of the FET at different gate voltages. All scale bars are 500 nm. (b) Simulated AC_MIM signal as a function of the 2D sheet conductance σ_{sh}. The insets show the quasi-static potential distribution at σ_{sh} = 10^{-10} and 10^{-3} S/□, respectively. Scale bar is 50 nm. The imaginary part of the signal monotonously increases with the sheet conductivity, while the real part of the signal reaches its maximum at $\sigma_{sh} \sim 10^{-7}$ S/□ then drops back to zero. The evolution of the MIM signals with respect to the gate voltages is consistent with the simulation result.

Supplementary Table

Supplementary Table 1. Comparison with previous work. Comparison of this work with other realistic solid-state CMOS inverters based on TMD channel materials.

Reference	Substrate (Gate Dielectric)	n-MOS/p-MOS	DC Gain	V_{IN} (V)	V_{DD} (V)
This work	Li-ion glass (Li-ion glass)	MoS_2/ WSe_2	34	0 ~ +1	1
Atiye et al., ACS NANO, 10, 2016	Glass (50 nm Al_2O_3)	MoS_2/ $MoTe_2$	33	0 ~ +1	1
June et al., ADVANCED MATERIALS, 29, 2017	Glass (50 nm Al_2O_3)	$MoTe_2$/ $MoTe_2$	18	0 ~ +1	1
Stefano et al., ACS NANO, 11, 2017	SiO_2/Si (12 nm h-BN)	$MoTe_2$/ $MoTe_2$	3	0 ~ +1	1
Mahmut et al., ACS NANO, 8, 2014	SiO_2/Si (20 nm ZrO_2)	WSe_2/ WSe_2	3	0 ~ +1	1
Yann-Wen et al., NANOSCALE HORIZONS, 4, 2019	SiO_2/Si (3 nm SiO_2+ 10 nm HfO_2)	MoS_2/MoS_2	16	0 ~ +2	2

Supplementary Notes

Supplementary Note 1. Speed of Li-ion glass back-gated TMD FET

The speed of EDLT device operation is mainly dependent on the timescale of EDL formation, where the timescale can be reduced by increasing ion conductivity and/or electric field strength. A Li ion conductivity of approximately 0.22 mS cm^{-1} was obtained at room temperature by using Nyquist plot (see Supplementary Fig. 6), which agrees well with the expected value in Li-ion glass;[3] ionic liquids, alternatively, have a conductivity on the order of 1 mS cm^{-1}.[4] From the low-amplitude AC impedance analyzer data in Fig. 1f, a complete/partial EDL formation is observed up to a frequency of ~ 100 kHz, similar to the observed timescale (up to a hundred kHz) in ionic liquid/gel.[5,6] The timescale for EDL formation can be experimentally improved further (~ MHz) by using a higher amplitude DC pulse (few mV nm^{-1}), limited by the electrochemical window (voltage at which oxidation and reduction reactions takes place in the electrolyte) of the electrolyte (> 4.3V for solid electrolytes). Even going beyond (few hundreds mV nm^{-1}) the electrochemical window with a substantially short period of time to avoid electrochemical reactions, was theoretically found to induce EDL in a much shorter timescale (GHz), technologically relevant for electronics applications.[7]

Supplementary Note 2. Drain current saturation in Li-ion glass back-gated TMD FET

For lower overdrives (V_{OV}=V_{BG}-V_{TH}), the current saturates due to channel pinch-off near the drain and $I_{D,sat}$ α V_{OV}^2, whereas for higher overdrives, carrier velocity saturates at lateral V_{DS} (and average lateral fields) lower than required to cause pinch-off and $I_{D,\upsilon sat}$ α $V_{OV}\upsilon_{sat}$. The crossover between the pinch-off and velocity saturation regimes will occur when the average lateral field $V_{OV}/L \approx 2\upsilon_{sat}/\mu_{eff}$.[8]

From the I_D-V_D output characteristics of MoS_2 FET (Supplementary Fig. 9), it is observed that the crossover between channel pinch-off and velocity saturation regions occurs approximately at an overdrive voltage of 0.6 V (V_{BG}=1.0V, V_{TH}=0.4V). Also, drain currents at saturation follows quadratic relationship with V_{OV} (channel pinch-off region) up to the crossover point (V_{BG} ~1.00V or V_{OV} ~ 0.6V) and after that it follows linear (constant spacing) relationship (velocity saturation region). Interestingly, unlike MoS_2, no crossover between pinch-off and velocity saturation regimes for saturation current in WSe_2 FETs has been observed, even with the application of a comparatively higher back-gate voltage (up to -1.4 V) (Supplementary Fig. 9). This dissimilarity between Li-ion back-gated MoS_2 and WSe_2 FETs can be attributed to a comparatively higher critical field for velocity saturation in the latter, which contrasts with a theoretical prediction of a higher critical field (E_{crit} α υ_{sat}/μ_{eff}) of the former.[9] This anomaly may be due to the strong effect of optical phonons in the Li-ion glass substrate on the channel transport, not considered in the theoretical work, and further elucidation on that requires a thorough and complex study of the substrate effect on the velocity saturation of various TMDs.

Supplementary Note 3. Unipolar conduction in Li-ion glass back-gated TMD FET

The contact resistance (R_c) with a Ni contact has been determined to be ~ 40 kΩ.µm at $n > 10^{13}$ cm^{-2} using four probe technique (shown in Supplementary Fig. 11), similar to the reported values (10-100 kΩ.µm) for a SiO_2/Si back-gated single layer CVD MoS_2 with the same metal contact under the same deposition condition (e-beam, ~10^{-6} Torr).[10] In contrast, several orders of magnitude decrease in contact resistance with ionic liquid top-gating over conventional SiO_2/Si back-gate has been achieved,[11] which is ascribed to the significant reduction of Schottky barrier (SB) width down to the order of electrostatic screening length of ionic liquid (~ 1 nm) caused by the strong electrostatic action of the ions (in liquid) close to metal/semiconductor interface at higher gate voltage.[12] The significant reduction in SB width facilitates enhanced carrier injection of both electrons and holes via tunneling through the SB, thereby realizing hole channel conduction even in the presence of a relatively high Schottky barrier height (SBH) of hole (~ 1 eV) in MoS_2 FET.[13] The evolution of hole branch due to strong band bending assisted by ionic liquid gating together with the inherently strong electron branch gives rise to ambipolar transport in ionic liquid top-gated MoS_2 FET. [14]

On the other hand, in back-gated solid electrolytic substrates, the ions are likely to be at least several screening lengths away from the metal/semiconductor interface since material and contact integration is not going to be as intimate as a liquid interface. In this case, band bending/SB narrowing will not be as significant as compared to ionic liquid. This, together with the negligible change in SBH for both electron ($\Phi_B \approx 0.18$ eV) and holes ($\Phi_B \approx 1$ eV) in pristine semiconductor/metal contact by gating,[15–17] results in desirable unipolar electron branch in MoS_2 FET as observed in this work, consistent with a previous report.[18]

Supplementary References

1. Li, H. *et al.* From Bulk to Monolayer MoS2: Evolution of Raman Scattering. *Advanced Functional Materials* **22**, 1385–1390 (2012).
2. Braga, M. H. *et al.* Extraordinary Dielectric Properties at Heterojunctions of Amorphous Ferroelectrics. *J. Am. Chem. Soc.* **140**, 17968–17976 (2018).
3. Nakajima, K., Katoh, T., Inda, Y. & Hoffman, B. Lithium ion conductive glass ceramics: properties and application in lithium metal batteries. in *Symposium on Energy Storage Beyond Lithium Ion: Materials Perspective* (2010).
4. Sato, T., Masuda, G. & Takagi, K. Electrochemical properties of novel ionic liquids for electric double layer capacitor applications. *Electrochimica Acta* **49**, 3603–3611 (2004).
5. Pudasaini, P. R. *et al.* Ionic Liquid versus SiO2 Gated a-IGZO Thin Film Transistors: A Direct Comparison. *ECS J. Solid State Sci. Technol.* **4**, Q105 (2015).
6. Choi, Y. *et al.* Proton-Conductor-Gated MoS2 Transistors with Room Temperature Electron Mobility of >100 cm2 V–1 s–1. *Chem. Mater.* **30**, 4527–4535 (2018).
7. Xu, K. *et al.* Pulse Dynamics of Electric Double Layer Formation on All-Solid-State Graphene Field-Effect Transistors. *ACS Appl. Mater. Interfaces* **10**, 43166–43176 (2018).
8. Smithe, K. K. H., English, C. D., Suryavanshi, S. V. & Pop, E. High-Field Transport and Velocity Saturation in Synthetic Monolayer MoS2. *Nano Lett.* **18**, 4516–4522 (2018).
9. Jin, Z., Li, X., Mullen, J. T. & Kim, K. W. Intrinsic Transport Properties of Electrons and Holes in Monolayer Transition Metal Dichalcogenides. *Phys. Rev. B* **90**, 045422 (2014).
10. English, C. D., Shine, G., Dorgan, V. E., Saraswat, K. C. & Pop, E. Improved Contacts to MoS2 Transistors by Ultra-High Vacuum Metal Deposition. *Nano Lett.* **16**, 3824–3830 (2016).

11. Perera, M. M. *et al.* Improved carrier mobility in few-layer MoS_2 field-effect transistors with ionic-liquid gating. *ACS Nano* **7**, 4449–4458 (2013).

12. Braga, D., Gutiérrez Lezama, I., Berger, H. & Morpurgo, A. F. Quantitative Determination of the Band Gap of WS_2 with Ambipolar Ionic Liquid-Gated Transistors. *Nano Lett.* **12**, 5218–5223 (2012).

13. Liu, H., Neal, A. T. & Ye, P. D. Channel Length Scaling of MoS_2 MOSFETs. *ACS Nano* **6**, 8563–8569 (2012).

14. Zhang, Y., Ye, J., Matsuhashi, Y. & Iwasa, Y. Ambipolar MoS_2 Thin Flake Transistors. *Nano Lett.* **12**, 1136–1140 (2012).

15. Giannazzo, F. *et al.* Impact of contact resistance on the electrical properties of MoS_2 transistors at practical operating temperatures. *Beilstein J Nanotechnol* **8**, 254–263 (2017).

16. Giannazzo, F. *et al.* Ambipolar MoS_2 Transistors by Nanoscale Tailoring of Schottky Barrier Using Oxygen Plasma Functionalization. *ACS Appl. Mater. Interfaces* **9**, 23164–23174 (2017).

17. Das, S., Chen, H.-Y., Penumatcha, A. V. & Appenzeller, J. High Performance Multilayer MoS_2 Transistors with Scandium Contacts. *Nano Lett.* **13**, 100–105 (2013).

18. Wu, C.-L. *et al.* Gate-Induced Metal–Insulator Transition in MoS_2 by Solid Superionic Conductor LaF_3. *Nano Lett.* **18**, 2387–2392 (2018).
